# Non-Covalent Interactions in Polymers

**DOI:** 10.3390/polym15051139

**Published:** 2023-02-24

**Authors:** Alexander S. Novikov

**Affiliations:** 1 Institute of Chemistry, Saint Petersburg State University, Universitetskaya Nab., 7/9, 199034 St. Petersburg, Russia; a.s.novikov@spbu.ru or novikov@itmo.ru; 2 Infochemistry Scientific Center, ITMO University, Kronverksky Pr., 49, bldg. A, 197101 St. Petersburg, Russia

## Abstract

Non-covalent interactions are one of the key topics in modern chemical science. These inter- and intramolecular weak interactions (e.g., hydrogen, halogen, and chalcogen bonds, stacking interactions and metallophilic contacts) have a significant effect on the properties of polymers. In this Special Issue, “Non-covalent interactions in polymers”, we tried to collect fundamental and applied research manuscripts (original research articles and comprehensive review papers) focused on non-covalent interactions in polymer chemistry and related fields. The scope of the Special Issue is very broad: we welcome all the contributions that deal with the synthesis, structure, functionality and properties of polymer systems involving non-covalent interactions.

Non-covalent interactions are one of the key topics in modern chemical science. These inter- and intramolecular weak interactions (e.g., hydrogen, halogen, and chalcogen bonds, stacking interactions and metallophilic contacts) have a significant effect on the properties of polymers. This Special Issue of *Polymers* entitled “Non-covalent interactions in polymers” aims to address the most recent progress in the rapidly growing field of non-covalent interactions in polymer chemistry and related fields. Both experimental and theoretical studies, fundamental and applied research, application of machine learning and artificial intelligence in studies of non-covalent interactions in polymers and any forms of manuscripts (for example, reviews, mini-reviews, full papers, short communications, technical notes, and highlights) are welcome for consideration. The scope of the Special Issue is very broad: we welcome all the contributions that deal with the synthesis, structure, functionality and properties of polymer systems involving non-covalent interactions. This Special Issue will address the following bullet-point topics: experimental studies of non-covalent interactions in polymers; theoretical modeling of supramolecular polymeric systems; application of machine learning and artificial intelligence in studies of non-covalent interactions in polymers; development of polymer materials (1D, 2D, 3D) via non-covalent interactions; databases of non-covalent polymers. We welcome researchers focused on polymer science and related topics to contribute their research to our Special Issue.

To date, there are many interesting publications in the field of fundamental studies of non-covalent interactions in polymers. In [1], authors observed that dihalomethanes CH_2_X_2_ (X = Cl, Br, I) can be co-crystallized with the isocyanide complexes *trans*-[MX^M^_2_(CNC_6_H_4_-4-X^C^)_2_] (M = Pd, Pt; X^M^ = Br, I; X^C^ = F, Cl, Br) to yield an extended series comprising fifteen X-ray structures of isostructural adducts featuring 1D metal-involving hexagon-like arrays linked via halogen bonding. In [2], the novel iodine-rich iodobismuthates(III), where the halometalate anionic fragments are linked by diiodine spacers into 1D- or 2D-supramolecular polymeric systems, were discovered. In [3], the authors observed that reactions of chlorotellurates(IV) and Br_2_ afford the formation of one-dimensional supramolecular complexes of general formula Cat_2_{[TeCl_6_](Br_2_)} (Cat = Me_3_N^+^, PyH^+^, 4-MePyH^+^ and 1-MePy^+^) where dibromine fragments are “trapped” by [TeCl_6_]^3−^ via specific Br⋯Cl interactions (halogen bonding). In [4], 1D iodine-rich iodobismuthates(III) with the highest fraction of “captured” I_2_ molecules in the halometalates network (for p-elements) were reported. In [5], thermally stable supramolecular polymers of chloroplumbate(IV) and chlorostannate(IV) with Cl_2_ linkers were reported. In [6], authors found that oxochloroselenate with incorporated Cl_2_ units, (tetramethylammonium)_3_{[Se_2_O_2_Cl_7_](Cl_2_)}, features very strong halogen bonding in solid state and high thermal stability. In [7], three polymeric group 11 transition metal polymers featuring metallophilic interactions were obtained directly via the self-assembly of metal ions and 4-pyridinethiol ligands. In [8,9], the influence of non-covalent interaction on the self-healing of mechanical properties in supramolecular polymers was discussed. In [10], non-covalent interactions in polymer–graphene nanocomposites and their effects on electrical conductivity were analyzed. In [11], core/shell conjugated polymer/quantum dot composite nanofibers through orthogonal non-covalent interactions were presented. In [12], the importance of CH···X (X = O, π) non-covalent interactions in Cu(II) coordination polymers was highlighted. In [13], the effect of molecular structure on the chain mobility of dichalcogenide-based polymers with self-healing capacity was analyzed. In [14], the influence of non-covalent contacts on photoluminescence properties of Cd and Cd-Ln pentafluorobenzoates with 2,2′:6′,2′-terpyridine derivatives was investigated. In [15], the effect of non-covalent interactions on the 2,4- and 3,5-dinitrobenzoate Eu-Cd Complex structures was discussed. In [16], chitosan functionalization via covalent and non-covalent interactions was reviewed. In [17], self-healing supramolecular hydrogels based on reversible physical interactions were introduced. In [18], surface plasmon resonance studies on molecular imprinting and the influence of non-covalent interactions on these processes were discussed. In [19], theoretical insight into the interaction between chloramphenicol and functional monomer (methacrylic acid) in molecularly imprinted polymers was introduced. In [20], advances in the multi-orthogonal folding of single polymer chains into single-chain nanoparticles were reviewed. In [21], enhancing the mechanical performance of a polymer material by incorporating pillar[5]arene-based host–guest interactions was highlighted. In [22], a combined molecular dynamics and DFT simulation study of the molecular and polymer properties of a catechol-based cyclic oligomer of polyether ether ketone was performed. In [23], chemomechanical polymers as sensors and actuators for biological and medicinal applications involving non-covalent interactions were discussed. In [24], various applications of supramolecular gels were reviewed. In [25], electrically self-healing thermoset composites based on Diels–Alder cycloaddition and hydrogen bonds were reported. In [26], copolymers and hybrids based on carbazole derivatives and their nanomorphology investigation were discussed. Finally, in [27], phytosterol recognition via rationally designed molecularly imprinted polymers and non-covalent interactions was highlighted.

I hope that other authors will follow my initiative and readers of this Special Issue of *Polymers* will have the opportunity to get acquainted with the achievements of researchers in this modern topic. 

## Data Availability

Not applicable.
